# Hemorrhagic Skull Base Chordoma Presenting As Chordoma Apoplexy

**DOI:** 10.7759/cureus.19187

**Published:** 2021-11-01

**Authors:** Ece Uysal, Michael A Cohen, Hussam Abou-Al-Shaar, Cheryl Palmer, William Couldwell

**Affiliations:** 1 Neurosurgery, University of Utah, Salt Lake City, USA; 2 Neurological Surgery, University of Pittsburgh Medical Center, Pittsburgh, USA; 3 Pathology, University of Utah, Salt Lake City, USA

**Keywords:** clivus, chordoma, skull base, hemorrhage, apoplexy

## Abstract

Intratumoral hemorrhage/apoplexy in clival chordomas is extremely uncommon, with only 16 reported cases. The average age of patients was 46.2 years and slightly more than half were men. In cases published before 1990, all patients died from their disease without any intervention. Since then, 11 patients have undergone resection by a variety of approaches and there have been no deaths. The diagnosis of skull base chordomas relies on a combination of clinical presentation and radiographic features related to the location and invasion of the tumor. Chordomas presenting with sudden-onset symptoms should alarm the surgeon of a possible hemorrhage. As an illustration of this presentation, we describe a 58-year-old woman who presented with acute-onset headache and cranial nerve deficits. Computed tomography and magnetic resonance imaging demonstrated a hemorrhagic clival lesion with cavernous sinus extension. The patient underwent transsphenoidal resection of the lesion that resulted in the resolution of her symptoms. Histopathological evaluation of the lesion was consistent with chordoma with acute hemorrhagic components. Although intratumoral hemorrhage is rarely detected in chordomas, it should be considered a differential diagnosis of such lesions because prompt recognition and treatment are critical for patient survival.

## Introduction

Skull base chordomas are aggressive recurrent intracranial lesions thought to originate from primitive notochord remnants and constitute <1% of all intracranial neoplasms [1] and 2%-4% of primary brain tumors [2]. Clival chordomas often remain asymptomatic while growing, causing extensive bony erosion before becoming symptomatic from local mass effect and resulting cranial neuropathy. Intratumoral hemorrhage/apoplexy in skull base chordomas is extremely rare and often results in acute presentation secondary to a sudden increase in mass effect and intracranial pressure. We review the literature and included a case illustration to emphasize that patient survival can be achieved with prompt recognition and surgery.

## Case presentation

A 58-year-old hypertensive woman with a remote history of breast cancer treated with mastectomy and chemotherapy presented to the emergency department with progressive left-sided acute-onset headache, nausea, vomiting, double vision, and left-sided partial ophthalmoplegia and ptosis of five-day duration. The neurological examination also demonstrated slight anisocoria consistent with partial left oculomotor nerve palsy. The left pupil was in the "down-and-out" position. Other neurologic findings were unremarkable. All pituitary hormones were within normal limits, except for a slightly elevated cortisol level (23.8 μg/dL, normal 6.0-23.0) and a decreased adrenocorticotropic hormone value (<5 pg/mL, normal 6-58).

**Figure 1:**
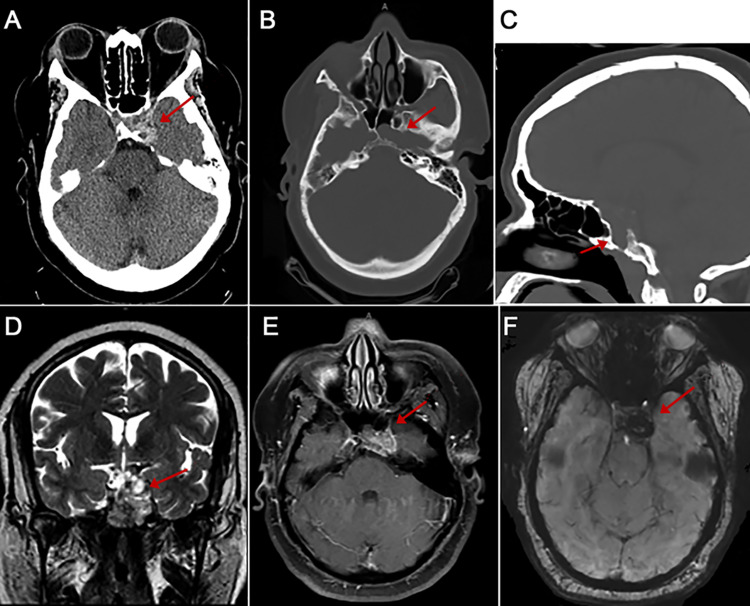
Imaging demonstrating hemorrhagic clival chordoma arising from the clivus. Computed tomography (CT) scans show the lesion (red arrows). (A, B) Axial images showing hyperdense lesion with extensive osseous erosion of the left sphenoid sinus and petrous bone. (C) Sagittal image demonstrating erosion of the sella and skull base. Magnetic resonance imaging demonstrating the lesion (red arrows). Axial coronal T2-weighted (D), axial T1-weighted with contrast (E), and axial gradient-recalled echo (F) magnetic resonance imaging demonstrating a heterogeneous enhancing hemorrhagic mass within the left cavernous sinus and sella and encasement of the cavernous internal carotid artery.

Computed tomography (CT) of the head demonstrated a high-density lesion arising from the clivus, with extensive osseous erosion into the left greater wing of the sphenoid and left petrous apex and scattered hyperdensities within the tumor bed (Figures 1A-1C). Magnetic resonance imaging (MRI) of the brain demonstrated a lobular mass arising from the clivus with extension into the left cavernous sinus, left petroclival fissure, and posterior aspect of the sphenoid sinus. The cavernous internal carotid artery (ICA) was encased with a tumor without narrowing or occlusion. The T2 hyperintense, heterogeneously enhancing mass measured 1.8×2.9×2.3 cm in diameter (Figures 1D-1F). Susceptibility-weighted imaging (SWI) also confirmed intratumoral hemorrhage. CT scans of the chest, abdomen, and pelvis revealed no signs of a primary oncologic process or metastatic disease.

The patient underwent a microscopic transsphenoidal approach with endoscopic assistance for excisional biopsy to establish a pathologic diagnosis and decompress the cavernous sinus. The left sphenoid sinus and clival recess were exposed. The tumor was removed from the left clival recess and followed into the left cavernous sinus, medial and lateral to the cavernous ICA. The cavernous component of the tumor was visualized and resected with 30° and 45° angled endoscopes. Once adequate decompression was achieved, hemostasis was obtained. Nasal packings were used for closure, and no cerebrospinal fluid leak was encountered.

**Figure 2:**
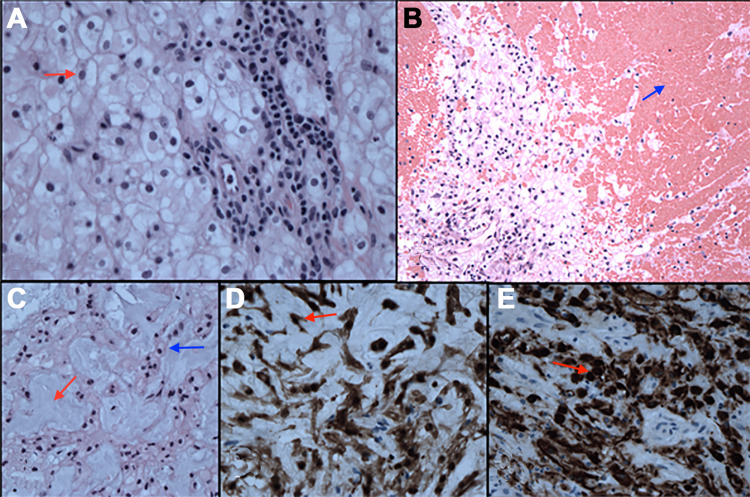
High-magnification hematoxylin and eosin (H&E) stain. High-magnification (A, C: ×400; B: ×200) H&E stain showing cellular tumor composed of plump cells (red arrow) with well-defined cytoplasmic borders (A), and organizing hemorrhage (blue arrow) (B), with myxoid matrix (red arrow) and cord-like arrangements (blue arrow) (C). High-magnification (×400) immunohistochemical analysis demonstrating focal positivity for S-100 protein (red arrow) (D) and epithelial membrane antigen (EMA), and diffuse positivity for broad-spectrum cytokeratins (red arrow) (E).

Histological examination revealed a moderately cellular tumor comprising plump cells with well-defined cytoplasmic borders (Figure 2A), foamy cytoplasm, and oval-to-irregular nuclei with minimal pleomorphism. Abundant acute and organizing hemorrhage was seen (Figure 2B). The cells were embedded in a myxoid matrix and focally present in cord-like arrangements (Figure 2C). Rare mitoses and focal necrosis with chronic inflammation were present. Immunohistochemical analysis revealed focal positivity for S-100 protein (Figure 2D) and epithelial membrane antigen and diffuse positivity for broad-spectrum cytokeratins (AE 1/3) (Figure 2E). These findings were consistent with a diagnosis of hemorrhagic chordoma.

The patient tolerated the procedure well with no complications. Postoperatively, her ophthalmoplegia and ptosis improved dramatically, with no new neurologic deficits. She was discharged home on postoperative day 3 and referred for proton beam radiotherapy. On telephone follow-up seven months after her surgery, she reported complete resolution of her symptoms and no new neurological issues.

## Discussion

Skull base chordomas are slow-growing, aggressive, recurrent tumors marked by extensive local invasion and destruction of surrounding structures [3]. Although acute intratumoral hemorrhage can be encountered [4], hemorrhagic transformation and apoplexy are exceedingly rare presentations of clival chordomas. Among the 17 reported cases of hemorrhagic clival chordoma, including the present case [5-20], the average age of patients presenting with spontaneous hemorrhage was 46.2 years (range 10-75 years). Ten (58.8%) patients were males, despite the typical predominance of skull base chordomas in females. The six cases published before 1990 all resulted in death without any interventions and were diagnosed on autopsy [5,6,16,17,19,20]. The remaining 11 patients underwent resection, with four purely endonasal, one combined endonasal and middle fossa, two frontotemporal, two transpetrosal, and two suboccipital approaches. There were no deaths among the 11 surgically treated patients [7-15,18].

Acute hemorrhage is most commonly intratumoral (75%) [5-15] but can involve the subarachnoid (17%) [9,16,17], intracerebral (17%) [6,15,16], and intraventricular (17%) [8,13,18] compartments. Hemorrhagic skull base chordomas were mostly seen in the clival region, but extension and invasion through adjacent structures have been reported in the sella [8,18], temporal lobe [11], sphenoid sinus [6,8,14], petrous bone [6,11,12,17], prepontine cistern [10,14], and midbrain [7,12,15]. One previous case of hemorrhagic cavernous sinus chordoma was reported in a pediatric patient [12]; our case of hemorrhagic chordoma showed rapid expansion into the cavernous sinus in an adult patient.

The diagnosis of skull base chordomas relies on a combination of clinical presentation, histopathologic, and radiographic features related to the location and invasion of the tumor. Chordomas presenting with sudden-onset symptoms should alarm the surgeon of a possible hemorrhage. Sudden headache (82.3%) [7,8,15,17] and vomiting secondary to increased intracranial pressure are the most common acute clinical presentations, but hemiparesis (23.5%) [7,13,15,17] and cranial nerve deficits - most commonly cranial nerve III (23.5%) [7,12,13] and VI (11.7%) [13,15] palsies - have also been reported. Diplopia (23.5%) [5,12,13], ptosis (11.7%) [12], facial numbness (5.8%) [15], and dysarthria (5.8%) [17] have also been encountered, and our patient had painful ophthalmoplegia and ptosis, suggesting that varied presentations are possible.

Skull base chordomas generally present as well-circumscribed, osteolytic, destructive lesions with associated cortical and lytic bone destruction and soft-tissue extension on non-contrast CT scans [12,21], which may also depict intratumoral calcifications. Clival chordomas generally have low-to-intermediate signal intensity on T1-weighted images and high intensity on T2 imaging, with moderate contrast enhancement [22]. The foci of hyperintensity on T1 may correlate with mucous or hemorrhage, and heterogeneous hypointensity on T2 may also be associated with mucous, hemorrhage, or calcifications. These hemorrhagic foci or calcifications should be confirmed with SWI or gradient recalled echo. The present case demonstrated mild erosion of the dorsum sellae and petrous bone on CT, and MRI demonstrated a T2-hyperintense lesion within the cavernous sinus with focal areas of T1 hyperintensity with corresponding T2 hypointensity and hypointensity on SWI consistent with areas of hemorrhage.

Macroscopically, chordomas are generally lobulated with a gelatinous matrix or chondroid solid features. Microscopically, chordomas take one of three forms. Chordoma not otherwise specified or classical chordoma is the most common form and is composed of lobules of epithelioid cells separated by septa of fibrous tissue in a myxoid matrix. The cells have vacuolated cytoplasm and are referred to as physaliphorous cells. Chondroid chordoma is the second histological form and comprises an extracellular matrix resembling hyaline cartilage. The last histological form is dedifferentiated chordoma, in which the tumor shows features of both classical chordoma type and undifferentiated spindle cell tumors [23]. This form is the most aggressive and is associated with a worse prognosis [24]. Chordomas are typically positive for cytokeratin, epithelial membrane antigen, S-100, and brachyury on immunohistochemical analysis [23].

Although the cause of hemorrhage remains largely unknown, various mechanisms have been proposed. Some authors have reported that rapid tumor growth without adequate matched blood supply may cause these small friable vessels to rupture [8]. Vascular proliferation and subsequent occlusion of smaller vessels may also cause tumor necrosis and hemorrhage. Other theories have suggested the clival chordomas in contact with the dura may cause dural vessels to proliferate, leading to hemorrhage [18].

Despite the importance of maximal surgical resection, adjuvant therapy remains an essential part of the armamentarium in the management of skull base chordoma, especially in cases where complete resection is not feasible [25]. Postoperative proton beam radiotherapy is the most commonly employed modality, with an average 5-year local tumor control and overall survival rates of 69.2% and 79.8%, respectively. In comparison, these rates are 36% and 53.5%, respectively, for conventional radiation therapy; 50% and 82%, respectively, for stereotactic fractionated radiation therapy; and 56% and 75%, respectively, for radiosurgery [26]. Chordomas are generally chemoresistant tumors, and chemotherapeutic agents are still under investigation in the management of these lesions [27].

## Conclusions

Hemorrhagic clival chordoma arising from the cavernous sinus is a rare clinical and radiographic presentation. The presence of skull base lesions with mixed T2 hyperintensity and focal areas of hemorrhage and bone erosion should raise a high index of suspicion for hemorrhagic clival chordoma, which presents acutely with apoplexy and resulting neurological deficits, requiring prompt recognition and treatment by skull base neurosurgeons for patient survival.
